# The Relationship Between Children’s Scale Error Production and Play Patterns Including Pretend Play

**DOI:** 10.3389/fpsyg.2020.01776

**Published:** 2020-07-22

**Authors:** Mikako Ishibashi, Izumi Uehara

**Affiliations:** Department of Psychology, Ochanomizu University, Bunkyo-ku, Japan

**Keywords:** scale error, toddler, play behavior, pretending, individual differences

## Abstract

Children of about 2 years of age occasionally make scale errors, e.g., they may attempt to fit their body into extremely small objects. Although previous studies have suggested that immature cognitive abilities may be responsible for these errors, the mechanism of scale error production is unclear. Because we assumed that obtaining characteristics of scale error behavior in the context of play would give us more useful indications concerning individual differences in producing scale errors, we examined how children engage in scale error behavior in relation to other types of play behavior, such as pretending, during the scale error task. The results indicate that children who produced scale errors exhibited less pretend play with miniature toys and tended to refuse to play with miniature toys more often than those who did not produce any scale errors during the task. Moreover, among the children who produced scale errors, the children who produced more scale errors were less likely to touch the miniature objects and less likely to perform pretending actions than those who produced fewer scale errors. These results suggest that pretense play is deeply related to a lower production, or no production, of scale errors. Some immature cognitive abilities underlining pretense play can be assumed to be related to the production of scale error. In conclusion, this study is one of the first to demonstrate empirically significant relationships between children’s scale error production and pretend behaviors, although further studies are necessary to understand the mechanisms.

## Introduction

Young children occasionally attempt to fit their body into extremely small objects. For example, they may attempt to wear very small shoes, as if they were putting on normal-sized shoes. This phenomenon is called “scale error” (DeLoache et al., 2004). “Scale error” is defined as children’s object-inappropriate behavior by misidentifying a miniature-sized object as normal in size without considering their own body-size (DeLoache et al., 2004; Brownell et al., 2007; Casler et al., 2011). Although this may appear to be pretense, this has recently come to be distinguished from pretending, i.e., children producing “scale error” seriously attempt to carry out that action, as evidenced by not expressing a “knowing smile” (DeLoache et al., 2013). Children’s scale errors are observed in situations, where children first freely interact with normal-sized toys and then are exposed to miniature-sized toys (DeLoache et al., 2004). This situational setting has recently come to be called the “scale error task.” Rosengren et al. (2010) examined individual differences in scale error production for children aged 18–29 months using this task.

Scale errors are observed not only in laboratory-based situations (Brownell et al., 2007; DeLoache et al., 2013) but also in home-based settings (Rosengren et al., 2009b). Rosengren et al. (2009a) observed children playing with miniature-sized toys during free play in preschool classrooms over a period of 3 months. Although nearly half of the children never produced scale errors, the toddler class children aged 17–28 months exhibited the greatest number of scale errors compared with children in the other two groups (an infant class of children aged 4–16 months and a 2-year-old class of children aged 29–40 months). There are two important points regarding these findings. First, some children never produce a scale error during their childhood. Second, if a child produces a scale error, the child tends to produce scale errors most at about 2.0 years of age and the child comes to exhibit no scale errors when they are 2.5–3.0 years of age.

The factors responsible for the production of scale errors were discussed in previous studies. The most frequently discussed idea concerning the underlying mechanism of scale errors has been that of immaturity with regard to integrating visual information appropriately into a series of actions (DeLoache et al., 2004). The details are as follows: children’s mental representation, which includes category information of normal-sized objects and the motor plan in relation to them, may be evoked when seeing a miniature object. The motor plan for the normal-sized object is usually inhibited by the visual representation of the object’s size, such that children select an appropriate action plan toward the miniature object. However, the immature visual representation of a miniature toy by children who exhibit scale error may not serve to inhibit the motor plan associated with a normal-sized toy (Ware et al., 2006). The other factors assumed were the child’s low sensitivity to size changes (Grzyb et al., 2017), immaturity regarding the concept of size (Ishibashi and Moriguchi, 2017), immaturity of their body-size awareness (Brownell et al., 2007), and/or misunderstanding of the function of the object (Casler et al., 2011).

Considering that even infants approaching the end of the first year of their life can grasp or reach for objects of different sizes and scale actions to objects appropriately (e.g., Fagard and Jacquet, 1996; Schum et al., 2011) and some children never produce a scale error during their childhood, these previous ideas do not explain the cause of scale error reasonably, and thus the mechanism remains controversial.

More basic practical questions, such as how a child produces scale error behavior, how long the child engages in scale error behavior during play in relation to other types of play behavior, and to what extent scale error behavior can be observed accompanying pretend behavior during the same play session, remain. Documenting the characteristics of scale error behavior more precisely in the context of play is necessary to develop the underlying mechanism for producing a scale error.

Anecdotal data indicate that behaviors differ between children with and without scale errors during the period immediately after the time when normal-sized objects are replaced by miniature objects (DeLoache and Uttal, 2011). Children who do not show a scale error change their behavioral patterns at once, interacting with miniature toys in appropriate ways depending on their sizes and features, such as pushing a miniature car on the floor. Children who exhibit a scale error do not change their behavioral pattern and interact with miniature objects in the same way as with normal-sized objects (DeLoache and Uttal, 2011). Rosengren et al. (2010) also supposed that comparing children’s scale error behaviors and other play behaviors would provide insight into the mechanism of scale error production; however, few empirical studies have provided any clarification regarding the differences in these behaviors.

The present study investigated the characteristics of scale error behavior in the context of play compared with other types of play behavior, including pretend behavior, which has often been questioned regarding its difference from scale error behavior. By seeing scale error behavior in the context of play, we should be able to distinguish whether or not children exhibit an additional type of play during the same play period and whether scale error behavior has some relations to other play behavior including pretend play. We should also be able to investigate whether scale error behavior is sequentially related with the specific behavior exhibited before or after the scale error behavior and whether or not short-time inhibition control is related to the production of scale error. If children cannot inhibit previous action toward the normal-sized object and then exhibit scale error behavior, then one would expect to observe scale error behavior more often in the beginning of the session than later in the session of the scale error task, in which the miniature object is replaced with a normal-sized object.

We will briefly review the development of behaviors toward objects, including scale error behavior and pretend behavior. Several studies have shown that children’s object-directed behaviors become more sophisticated and elaborate with age (McCarty et al., 1999; Sommerville and Woodward, 2005). Considering the significant increase in pretend play after the first year of age, Power (2000) supposed that the quality of play should change along with the increase in pretend play. Lillard et al. (2013) argued that children’s symbolic play with an object should be distinguished from a real action toward the object in that symbolic play requires inhibiting inappropriate actions, i.e., real actions should be inhibited during the period when children are playing with the objects regarding them “as-if” they were real. Rosengren et al. (2010) speculated that the developmental decrease in scale errors in children may be related to the increase in pretend play. Accordingly, a longitudinal study by He et al. (2015) reported that children who showed scale errors were less likely to express scale error 11 months later, when pretend behavior occurs more frequently than before.

However, to our knowledge, no study has examined the details of behaviors by children who produced and did not produce scale errors in relation to pretend play, during the same play period. Considering the above results by He et al. (2015), frequencies of scale error exhibition and pretense play might be placed in the developmental course of children’s play patterns. Based on the literature, we can say that pretend play reflects intentional and flexible manipulation of representations accompanied by a positive feeling (Friedman and Leslie, 2007; Lillard et al., 2013; Carlson et al., 2014). In contrast, scale error behavior is often accompanied by a negative reaction (Ware et al., 2010) as there is less flexibility.

In light of these considerations, scale error may be the result of a failure to select other behaviors, such as pretending. It can be assumed that children who do not produce a scale error can be characterized as producing more sophisticated appropriate behavior, including pretend behavior. Therefore, the present study examined the details of children’s behavior during the scale error task and focused on the frequency of scale error and pretend behaviors.

The purpose of this study was to examine children’s behavior, in addition to scale error behavior, with miniature play objects during the scale error task, to clarify whether children with and without scale errors respond similarly or differently toward the objects. We can confirm whether scale error behavior is transiently observed immediately after the change in toy size or when miniature toys are presented by checking whether or not short-term inhibitory control is involved in the production of scale error. We assumed that the children who exhibited a scale error even when real normal-sized toys were replaced with miniature toys, would hardly exhibit different and/or specific play patterns toward miniature toys, such as pretending and flexibility in their play. In contrast, children without scale error would exhibit different play patterns, including pretending that the miniature toys were real but not true, real-sized toys, because they could intentionally manipulate the representations and thus could play with the toys more flexibly.

## Materials and Methods

### Participants

In total, 75 typically developing children [mean age (*M*) = 21.75 months, standard deviation (*SD*) = 4.93 months; 32 girls and 43 boys] between 15 and 35 months of age participated in our study. The age range was determined based on previous studies that investigated children’s scale error (DeLoache et al., 2004; Ware et al., 2006). Eleven additional children were excluded due to (1) fussiness and/or (2) refusal to interact with toys from the beginning of the session.

All participants were recruited *via* flyers placed in various places (i.e., nurseries and libraries) or by e-mail invitations from the database of the developmental lab at our university. They lived in Tokyo or in the vicinity and were from middle-class backgrounds. Both verbal consent and written informed consent for the children to participate were provided by the parents. In accordance with the Declaration of Helsinki, the procedures of the present study were approved by the Humanities and Social Sciences Research Ethics Committee of Ochanomizu University.

### Materials

#### Scale Error Task

Stimuli were comprised of four types of toy objects: a slide, a car, a chair (the chair was accompanied by a desk, and a book was on the desk), and a pair of shoes. Each object had both child-sized and miniature-sized versions, except for the shoes. The shoes were those that children took off to enter the playroom immediately before the task, and the miniature-sized shoes were slightly different in color and design from their shoes. The sizes of the objects were as follows: slide, 46.0 × 110.0 × 72.0 and 5.0 × 21.0 × 14.0 cm^3^; car, 58.0 × 37.5 × 35.5 and 7.0 × 6.0 × 10.0 cm^3^; desk, 61.0 × 41.0 × 47.5 and 10.0 × 18.0 × 15.0 cm^3^; chair, 35.5 × 28.5 × 32.0 and 8.0 × 10.0 × 10.0 cm^3^; book, 12.0 × 17.0 and 2.0 × 3.0 cm^2^; and shoes, 4.0 × 7.0 × 2.0 cm^3^ (miniature only). Two video cameras were used for the data analysis of the children’s behaviors.

### Procedure

The scale error task was conducted in the play space of a university playroom after the child had become accustomed to the room and the researcher and was relaxed. This warm-up time was 15–20 min. An outline of the task procedure is as follows:

*Phase 1*: The child was provided with only child-sized toys (a slide, a car, and a desk set) and was allowed to play with them freely for approximately 5 min.
*Phase 2*: The child and parent left the playroom for about 3 min. The three child-sized toys were replaced with their miniature versions and miniature shoes were added by the experimenter.
*Phase 3*: The child and the parent reentered the playroom. The child was asked to play again. The child’s behavior was observed for 5 min.
*After the task*: The child took a break. The parent completed the Japanese version of MacArthur-Bates Communicative Development Inventories (Watamaki and Ogura, 2004). The experimenter confirmed typical development in all participants.

The parent was instructed not to mention the size of the toys to the child and not to tell the child how to play with the toys, but was allowed to communicate freely with the child during the task. To prevent the experimenter’s words from having an influence on the child’s behavior, the experimenter only instructed the child to play with the toys at the start of phases 1 and 3, except for encouraging the child to play with the toys, such as the words, “how about this” and “do you want to play with this toy?” when the child did not show any interest in playing with the toys for some time after starting the phase. Such cases were few.

Task-onset latency was calculated for each child from the time when the child entered the play space to the time when the child began playing with the miniature objects. The duration of the miniature toy play session was analyzed only for the first 3 min from the child’s entrance into the play space. This is because five children left the playroom and had ceased playing without starting to play again within the 5-min observation period.

All play episodes during the scale error task session were annotated using ELAN version 5.0 software. This software allowed us to calculate the duration and count during the play session (Tacchetti, 2012). The detailed criteria for coding these behaviors are described below.

### Data Coding and Analysis

#### Children’s Scale Error

The coding criteria for the children’s scale error followed those set by DeLoache et al. (2013): (a) A child’s behavior toward the miniature-sized objects was the same as that toward the normal-sized objects. For example, the child put their foot on the miniature ladder. (b) The child’s appropriate body part(s) for touching the appropriate part(s) of the normal object also touched the corresponding part(s) of the miniature object. (c) Their effort or attitude toward the miniature object was serious. A five-point scale was used to judge the degree of seriousness (1: definitely serious–5: definitely pretending); selecting “1” or “2” was regarded as meeting criterion (c). The child whose seriousness was judged as “1” or “2” genuinely attempted to use the miniature toy in the way that the child had used the normal-sized toy. We regarded the behavior as a scale error if these three criteria were satisfied.

To enable the coders to judge the behaviors more easily, detailed examples were also included in the instruction paper about the coding of scale error, in addition to the coding criteria discussed above: “trying to climb into a miniature car” is scale error (Rosengren et al., 2009b), “serious, persistent and effortful attempt” is scale error (Rivière et al., 2020), and “pushing the car around on the floor, accompanied with improvised car noises” is pretending (DeLoache et al., 2004). About 25% of the data were coded by one of the authors and an independent psychological researcher (secondary coder); inter-rater reliability was good (*κ* = 0.74). Classification data that were disputed were solved through discussion. The remaining data were coded solely by the author.

#### Children’s Play Behaviors During the Scale Error Task Session

The other play behaviors, beside the scale error behaviors during the scale error task, were also classified. The children’s behaviors in relation to the miniature toys were checked first to determine whether they were scale error behaviors. If not, we then checked whether they were included in one of the four categories. The categories were the modified categories of DeLoache et al. (2013; Experiment 1) according to the purposes of this study. In our study, we did not include “no response” (ignorance of the experimenter’s prompt), because the experimenter’s prompt was not a crucial factor in the present study. In addition, “explicit refusal” (children’s verbal rejection) was changed to “refusal,” which was not restricted to verbal rejection but also included refusal to play with the miniature toys, because behavioral rejection could have some implications in the present study. The content of “other” (general exploration not including pretense) was replaced with “touching” to clarify the behavioral pattern.

Thus, the present categories were: (a) “standard pretense” referring to pretense play, such as pushing a car on the floor to run fast or letting their hands or toys go down the slide as in DeLoache et al. (2013); (b) “non-pretense play” referring to usual play except for pretense and scale error behaviors, such as putting the car on the chair or slide as in DeLoache et al. (2013); (c) “touching” referring to only touching the toys; and (d) “refusal” referring to the child not engaging or playing with the miniature toys, such as ignoring the miniature toys immediately after seeing them, leaving the miniature toys and the play space and rushing to the shelf outside of the play space to play with the other objects. The other responses not included in the four categories were pointing at the toys and keeping still (i.e., doing nothing).

To help the coders identify “standard pretense” more easily from scale error behavior, the instruction paper about the coding of play behaviors during the task included detailed examples, as mentioned above. Before coding play behaviors, it was confirmed that the children who engaged in “standard pretense” seemed to be enjoying the play, i.e., exhibited a positive demeanor (Lillard et al., 2013); in contrast, children tended to exhibit some negative reactions (i.e., upset, frustrated, confused, and surprised) when committing scale error (Ware et al., 2010). About 20% of the video recordings were coded both by a naïve researcher who was blinded to the purpose of the study and one of the present authors. The kappa coefficient between the two coders was excellent (*κ* = 1.00). The remaining data were coded by the author.

In the analysis, we used the proportion data of duration to perform statistical analyses under the conditions meeting a normal distribution of data and variance homogeneity like other studies (Rubin et al., 1978; Pellegrini and Gustafson, 2005; Pellegrini and Hou, 2011). To compare the ratio of each behavioral category other than scale error behavior between the scale error (SE) and no-scale error (NSE) groups, the proportions of the four types of play behavioral responses were compared between the two groups based on the duration of each categorical behavior divided by the duration of total time of the session, after subtracting the duration of scale error behavior. We also examined which behavior explained scale error according to the number of such errors, using the proportional data of the four classified behaviors.

## Results

Main analyses were conducted using R version 3.2.2; supplementary analyses were carried out with IBM SPSS version 25. Of the 75 children, 34 exhibited scale errors. Thus, 34 children were assigned to the SE group, and the remaining 41 children were placed in the NSE group. A two-tailed independent *t*-test revealed no significant difference in age between the two groups [SE group: *M* = 21.44, *SD* = 4.55; NSE group: *M* = 22.00, *SD* = 5.27; *t*(73) = 0.49, *p* = 0.63, *d* = 0.11, 95% confidence interval (CI; −1.73, 2.85)]. No significant gender differences were found in the ratios of children in the SE and NSE groups [*χ*^2^(1) = 0.49, *p* = 0.64]. The mean number of scale errors was 1.00 (*SD* = 1.39). No significant gender differences in the number of scale errors were observed [girls: *M* = 1.31, *SD* = 1.67; boys: *M* = 0.77, *SD* = 1.09; *t*(49.39) = −1.61, *p* = 0.11, *d* = 0.40, 95% CI (−1.23, 0.14)], and no significant correlation between age and number of scale errors was found (*r* = 0.01, *p* = 0.96). Therefore, we combined girl and boy data for further analysis; age was not included as a meaningful variable in the analysis.

First, to clarify whether there were critical differences in play behavior during the task between the SE and NSE groups of children, we conducted a two-tailed independent *t*-test for each variable. No significant difference was observed in onset latency between the two groups [SE group: *M* = 7.86, *SD* = 7.43; NSE group: *M* = 9.28, *SD* = 14.98; *t*(73) = 0.50, *p* = 0.62, *d* = 0.12, 95% CI (−4.20, 7.04)], which ensured no significant difference in performance level between the two groups. Table 1 describes the mean proportions of the four types of responses by the two groups. A two-tailed independent *t*-test revealed that children who did not exhibit a scale error (NSE group children) were significantly more likely to engage in “standard pretense” [*t*(73) = 2.82, *p* = 0.01, *d* = 0.65, 95% CI (−0.04, 0.25)]. No significant differences in “non-pretense play” or “touching” were found between the two groups [non-pretense play: *t*(73) = 0.54, *p* = 0.59, *d* = 0.13, 95% CI (−0.03, 0.05); touching: *t*(73) = 0.89, *p* = 0.38, *d* = 0.21, 95% CI (−0.05, 0.13)]. A marginal difference in “refusal” was observed between the two groups, indicating that the SE group were more likely to refuse to play with the miniature objects than the NSE group [refusal; *t*(44.11) = −1.88, *p* = 0.07, *d* = 0.47, 95% CI (−0.13, 0.01)].

**Table 1:** Children’s mean proportion of each behavioral category during the scale error task period, except for the time exhibiting scale error behavior.

	SE group (*N* = 34)		NSE group (*N* = 41)	
Children’s responses	*Mean*	*SD*	*Mean*	*SD*
Standard pretense	0.21	0.20	0.35	0.24
Non-pretense play	0.04	0.07	0.05	0.09
Touching	0.31	0.18	0.35	0.22
Refusal	0.10	0.18	0.03	0.08

SE, scale error; NSE, no-scale error; SD, standard deviation.

Next, we examined whether the number of scale errors could be explained by the children’s response to the miniature objects. Thus, the proportions of children’s responses of each type (standard pretense, non-pretense play, touching, and refusal) were included in multiple regression analyses as an independent variable to explain the number of scale errors. The variance inflation factor (VIF) for each of the four independent variables was <4, i.e., a stringent index value for multicollinearity (O’Brien, 2007) and all absolute values of the correlation coefficients (|*r*|) between variables were <0.5 (Dormann et al., 2013), indicating that there was almost no risk of multicollinearity for conducting this multiple regression analysis (Tables 2 and 3).

**Table 2:** Correlation between the proportions of standard pretense, non-pretense play, touching, and refusal.

Dependent variables	Standard pretense	Non-pretense play	Touching	Refusal
Standard pretense		0.05	−0.39^***^	−0.40^***^
Non-pretense play			−0.04	−0.05
Touching				−0.22^*^

*N* = 75.

* *p* < 0.05;

*** *p* < 0.001.

**Table 3:** Coefficients of “standard pretense,” “non-pretense play,” “touching,” and “refusal” in multiple regression analyses and the variance inflation factor (VIF).

Effect	B	*SE*	**β**	*t*	*p*	*VIF*
Intercept	3.32	0.54		6.16	<0.001^***^	
**Standard pretense**	−**3.66**	**0.79**	−**0.623**	−**4.64**	<**0.001** ^***^	**1.88**
Non-pretense play	−3.09	1.74	−0.175	−1.77	0.08	1.01
**Touching**	−**3.32**	**0.88**	−**0.481**	−**3.80**	<**0.001** ^***^	**1.67**
Refusal	−0.86	1.25	−0.086	−0.69	0.49	1.62

Note: *N* = 75; Bold values represent statistically significant effects except for intercept.

*** *p* < 0.001. VIF, variance inflation factor.

This regression model explained 33.0% of the variance [*F*(4,70) = 8.49, *p* < 0.001, adj. *R*^2^ = 0.29]. The summary of coefficients of this model is shown in Table 3. Table 3 illustrates that the proportions of “standard pretense” and “touching” were significant variables explaining the number of scale errors. The negative significant values of these variables meant that the longer the time duration of “standard pretense” and “touching”, the fewer the number of scale errors and vice versa.

Additionally, we checked to see what behaviors children exhibited initially (at the beginning of the session), and then the behaviors demonstrated subsequently over the course of the session when miniature-sized objects were provided, to see whether the children who engaged in scale error produced scale errors more often at the earliest time (first time) than at later times during the session. Table 4 lists the frequencies of the two frequent types of responses (“standard pretense” and “touching”), as well as “scale error” and “Other” (“Other” included “non-pretense play,” “refusal,” and other responses). We did not find significantly more frequencies of “scale error” at the earliest (first) time than at later times for the SE group. Specific play sequence patterns for SE and NSE groups could not be resolved. We could only confirm significant differences between the two groups in frequency of “scale error” and “standard pretense” [*χ*^2^(15) = 46.63, *p* = 0.00, *φ* = 0.46].

**Table 4:** Frequency of each play (standard pretense, touching, scale error, and the other) at the first, second, and third times from the start of the task session for children in SE and NSE groups.

	SE group (*N* = 34)			NSE group (*N* = 41)		
	First	Second	Third	First	Second	Third
Standard pretense	7	5^†^	10	18^*^	12	12
Touching	23	18	13^*^	23	26	26
Scale error	4	9^**^	9^**^	0^*^	0^*^	0^*^
Other	0	2	2	0	3	3
Total	34	34	34	41	41	41

SE group (*N* = 34), NSE group (*N* = 41), Other: includes non-pretense play, refusal, and other responses.

Residual analysis: ^†^*p* < 0.1;

* *p* < 0.05;

** *p* < 0.01.

## Discussion

The purpose of this study was to investigate the relationship between scale error production and play patterns during the scale error task. We hypothesized that children who do not produce any scale errors may exhibit other play behaviors, such as pretending, more frequently than those who produced a scale error during the scale error task.

Accordingly, the results revealed that children who did not produce any scale errors exhibited significantly more pretense play than those who produced a scale error, whereas the children who exhibited a scale error tended to refuse to play with the miniature toys. The data of the children who produced one or more scale errors indicated that children who produced more scale errors were less likely to touch the miniature objects and less likely to perform pretending actions than those who produced fewer errors. Supplementary analyses revealed that scale error behavior can be observed not only immediately after the child first discovers the miniature object but also at later times in the session, indicating that scale errors are produced when the child finds miniature toys without seeing the normal-sized ones.

Now, we discuss these results in relation to the explanation offered most often regarding the underlying mechanism of scale error, i.e., immaturity with regard to integrating visual information appropriately into a series of actions (e.g., DeLoache et al., 2004). According to this idea, it has been assumed that scale errors are produced when a child fails to inhibit their representation toward a normal-sized object (Ware et al., 2006) and/or that selecting an appropriate action is closely associated with inhibitory control ability (Munakata, 2001), i.e., uninhibited activated representation of normal-sized objects and thus inappropriate action toward the miniature objects. As indicated in our results, less pretense play by the children who presented scale errors than by those who did not provide partial support for this idea; here, less pretense may mean failure to select an appropriate play action, such as pretending, in relation to the miniature objects. The results from our supplementary analyses also suggest that scale errors are not caused by short-time fails or a delay in inhibition control, i.e., not being a perseverative uninhibited previous response. This may support the idea of immaturity in cognitive ability, such as continued failure in inhibiting previous representations, accompanied by an inappropriate action.

However, the lack of a significant difference in the proportions of other possible appropriate responses toward the miniature objects, such as non-pretense play, between the SE and NSE groups, as well as the regression results concerning an increase in the number of scale errors related to reductions in pretense and touching, cannot be explained solely by this idea. The fact that some children never produce a scale error during their childhood does little to support the idea of immaturity in ability, usually assumed to develop similarly among children. More refusal actions by the present children who produced a scale error may mean that they might have been perplexed by the miniature objects, realizing the size change to some extent, thus causing an inhibition to certain responses to the miniature objects while, at the same time, not being able to play appropriately with the miniature objects.

The present results also cannot be entirely explained by other ideas, such as children’s poor sensitivity to size changes (Grzyb et al., 2017), immaturity in relation to the concept of size (Ishibashi and Moriguchi, 2017), and immaturity in body-size awareness (Brownell et al., 2007), together with the fact that some children never produce a scale error during their childhood. The mechanism of producing scale error cannot depend solely on the child’s development of size awareness and sensitivity.

An interesting perspective was provided by Casler (2014), who reported that children at about 2 years of age are relatively persistent in “functional fixedness,” i.e., regarding one object as having only one function. Casler (2014) argued that size information may be neglected when children focus more on functional information. Considering that pretense is based on the understanding that an object has another meaning, less pretense play by children with scale error than by those with no scale error can be regarded as being related to persistent “functional fixedness.” The regression result that shorter durations of “standard pretense” and “touching” result in greater scale error may also be attributable to the persistence of “functional fixedness.” However, this idea hardly explains the other results, specifically, the lack of a significant difference in the proportions of other types of responses to the miniature objects between SE and NSE groups.

Next, we examine the present results focusing on the relationship between scale error behavior and pretense behavior. The scale error behaviors observed together with the other types of behavior during the play session, including pretend play, suggest that this phenomenon is related to play style. The two forms of play, scale error behavior and pretend behavior, were negatively related in frequency. Considering the nature of pretense play described in previous studies, i.e., intentional and flexible manipulations of representations enjoyed in play (Friedman and Leslie, 2007; Lillard et al., 2013; Carlson et al., 2014) and the scale error behavior often accompanied by negative reactions (Ware et al., 2010), scale error production might be one of the specific play styles used by children aged around 2 years who have fewer play patterns; this may lead to less frequent pretense play and refusal to play with miniature toys. Meanwhile, children who produce no scale errors might have more play patterns, including those involving advanced pretense and, thus, may respond flexibly. The fact that some children never exhibit scale errors during childhood could then be explained more reasonably by this notion of individual differences in the play patterns available to children of this age than by previous ideas. Moreover, this notion may provide more useful clinical implications than previous ideas such that guiding the children to produce more play patterns would stimulate their representational skills, which are related with cognitive and linguistic developments.

Although the findings of the present study suggest new ways to interpret the production mechanism of scale error, several limitations must be considered. To interpret the production of scale error from the developmental perspective of play style, more research using a larger sample including a wider range of early-childhood ages will be needed. That is, developmental changes related to play should examine whether there are developmental differences between having a few simple play patterns that sometimes produce scale errors and having more flexible play patterns that might lead to a variety of pretend play patterns. Detailed examination and comparison of not only frequencies but also of the contents of play behaviors, especially pretend play, using more variables among children with no, few, or many scale errors over a longer observation time than the present task time will be necessary to identify the underlying mechanism of scale error production in relation to other play behaviors.

In conclusion, this study is one of the first to demonstrate empirically significant relationships between children’s ways of playing with miniature objects, particularly pretend behaviors, and the number of scale errors in a between-subjects study design. These findings are expected to prompt further study of the developmental processes related to the occurrence and diminishment of scale error in the context of cognitive development.

## Data Availability Statement

Photo and coding schema in the scale error task, and datasets generated for this study are available on request to the corresponding author.

## Ethics Statement

In accordance with the Declaration of Helsinki, the procedures of the present study were approved by the Humanities and Social Sciences Research Ethics Committee of Ochanomizu University (Ethics approval number: 2016–1, 2017-109). Both verbal consent and written informed consent for the children to participate were provided by the participants’ legal guardian/next of kin.

## Author Contributions

MI and IU contributed substantially to the concept and design of the study and to the acquisition, analysis, and interpretation of data, drafting of the manuscript and revising it critically for important intellectual content. All authors contributed to the article and approved the submitted version.

## Funding

This research was supported by JSPS KAKENHI (grant number: JP18H05524).

## Conflict of Interest

The authors declare that the research was conducted in the absence of any commercial or financial relationships that could be construed as a potential conflict of interest.
