# Corrigendum: The relationship between children's scale error production and play patterns including pretend play

**DOI:** 10.3389/fpsyg.2022.1040479

**Published:** 2022-10-11

**Authors:** Mikako Ishibashi, Izumi Uehara

**Affiliations:** Department of Psychology, Ochanomizu University, Bunkyo-ku, Japan

**Keywords:** scale error, toddler, play behavior, pretending, individual differences

In the original article, there was an error. The authors reported the number of participants was 76, however, the actual sample size was 75. Due to the change in sample size, corrections to the statistical values were also required.

A correction has been made to *Materials and Methods, Participants, Paragraph 1*. The corrected paragraph is shown below.

In total, 75 typically developing children (mean age, *M* = 21.75 months, standard deviation, *SD* = 4.93, 32 girls and 43 boys) between 15 and 35 months of age participated in our study. The age range was determined based on previous studies that investigated children's scale error (DeLoache et al., 2004; Ware et al., 2006). Eleven additional children were excluded due to (1) fussiness and/or (2) refusal to interact with toys from the beginning of the session.

Corrections have also been made to *Results, Paragraphs 1 and 2*. The corrected paragraphs are shown below.

Main analyses were conducted using R version 3.2.2; supplementary analyses were carried out with IBM SPSS version 25. Of the 75 children, 34 exhibited scale errors. Thus, 34 children were assigned to the SE group, and the remaining 41 children were placed in the NSE group. A two-tailed independent *t*-test revealed no significant difference in age between the two groups [SE group: *M* = 21.44, *SD* = 4.55; NSE group: *M* = 22.00, *SD* = 5.27; *t*(73) = 0.49, *p* =0.63, *d* = 0.11, 95% confidence interval (CI) (−1.73, 2.85)]. No significant gender difference was found in the ratios of children in the SE and NSE groups [χ^2^(1) = 0.49, *p* =0.64]. The mean number of scale errors was 1.00 (*SD* = 1.39). No significant gender difference in the number of scale errors was observed [girls: *M* = 1.31, *SD* = 1.67; boys: *M* = 0.77, *SD* = 1.09; *t*(49.9) = −1.61, *p* = 0.11, *d* = 0.40, 95% CI (−1.23, 0.14)], and no significant correlation between age and number of scale errors was found (*r* = 0.01, *p* = 0.96). Therefore, we combined girl and boy data for further analysis; age was not included as a meaningful variable in the analysis.

First, to clarify whether there were critical differences in play behavior during the task between the SE and NSE groups of children, we conducted a two-tailed independent *t*-test for each variable. No significant difference was observed in onset latency between the two groups [SE group: *M* = 7.86, *SD* = 7.43; NSE group: *M* = 9.28, *SD* = 14.98; *t*(73) = 0.50, *p* = 0.62, *d* = 0.12, 95% CI (−4.20, 7.04)], which ensured no significant difference in performance level between the two groups. Table 1 describes the mean proportions of the four types of responses by the two groups. A two-tailed independent *t*-test revealed that children who did not exhibit a scale error (NSE group children) were significantly more likely to engage in “standard pretense” [*t*(73) = 2.82, *p* = 0.01, *d* = 0.65, 95% CI (0.04, 0.25)]. No significant differences in “non-pretense play” or “touching” were found between the two groups [non-pretense play; *t*(73) = 0.54, *p* = 0.59, *d* = 0.13, 95% CI (−0.03, 0.05), touching; *t*(73) = 0.89, *p* = 0.38, *d* = 0.21, 95% CI (−0.05, 0.13)]. A marginal difference in “refusal” was observed between the two groups, indicating that the SE group were more likely to refuse to play with the miniature objects than the NSE group [refusal; *t*(44.11) = −1.88, *p* = 0.07, *d* = 0.47, 95% CI (−0.13, 0.00)].

A correction has also been made to *Results, Paragraphs 4 and 5*. The corrected paragraphs are shown below.

This regression model explained 33.0% of the variance [*F*(4, 70) = 8.49, *p* < 0.001, adj. *R*^2^ = 0.29]. The summary of coefficients of this model is shown in Table 3. Table 3 illustrates that the proportions of “standard pretense” and “touching” were significant variables explaining the number of scale errors. The negative significant values of these variables meant that the longer the time duration of “standard pretense” and “touching,” the fewer the number of scale errors and vice versa.

Additionally, we checked to see what behaviors children exhibited initially (at the beginning of the session), and then the behaviors demonstrated subsequently over the course of the session when miniature-sized objects were provided, to see whether the children who engaged in scale error produced scale errors more often at the earliest time (first time) than at later times during the session. Table 4 lists the frequencies of the two frequent types of responses (“standard pretense” and “touching”), as well as “scale error” and “Other” (“Other” included “non-pretense play,” “refusal,” and other responses). We did not find significantly more frequencies of “scale error” at the earliest (first) time than at later times for the SE group. Specific play sequence patterns for SE and NSE groups could not be resolved. We could only confirm significant differences between the two groups in frequency of “scale error” and “standard pretense [χ^2^(15) = 46.63, *p* = 0.00, ϕ = 0.46].

Due to the error in sample size, corrections were also required to Tables 1–4 and their captions. The corrected tables and captions are shown below.

**Table 1:** Children's mean proportion of each behavioral category during the scale error task period, except for the time exhibiting scale error behavior.

**Children's responses**	**SE group (*****N*** = **34)**		**NSE group (*****N*** = **41)**	
	***Mean***	***SD***	***Mean***	***SD***
Standard pretense	0.21	0.20	0.35	0.24
Non-pretense play	0.04	0.07	0.05	0.09
Touching	0.31	0.18	0.35	0.22
Refusal	0.10	0.18	0.03	0.08

SE, scale error; NSE, no scale error; SD, standard deviation.

**Table 2:** Correlation between the proportions of standard pretense, non-pretense play, touching, and refusal.

**Dependent variables**	**Standard pretense**	**Non-pretense play**	**Touching**	**Refusal**
Standard pretense		0.05	−0.39^***^	−0.40^***^
Non-pretense play			−0.04	−0.05
Touching				−0.22^*^

*N* = 75,

* *p* < 0.05,

*** *p* < 0.001.

**Table 3:** Coefficients of “standard pretense,” “non-pretense play,” “touching,” and “refusal” in multiple regression analyses and the variance inflation factor (VIF).

**Effect**	**B**	***SE***	**β**	***t***	***p***	***VIF***
Intercept	3.32	0.54		6.16	< 0.001^***^	
**Standard pretense**	**−3.66**	**0.79**	**−0.623**	**−4.64**	< 0.001^***^	1.88
Non-pretense play	−3.09	1.74	−0.175	−1.77	0.08	1.01
**Touching**	**−3.32**	**0.88**	**−0.481**	**−3.80**	< 0.001^***^	1.67
Refusal	−0.86	1.25	−0.086	−0.69	0.49	1.62

*N* = 75; Bold values represent statistically significant effects except for intercept.

*** *p* < 0.001. VIF, variance inflation factor.

**Table 4:** Frequency of each play (standard pretense, touching, scale error, the others) at the first, second, and third times children of SE and NSE groups did from the start of task session.

	**SE (*****N*** = **34)**			**NSE (*****N*** = **41)**		
	**1st**	**2nd**	**3rd**	**1st**	**2nd**	**3rd**
Standard pretense	7	5^†^	10	18^*^	12	12
Touching	23	18	13^*^	23	26	26
Scale error	4	9^**^	9^**^	0^*^	0^*^	0^*^
The others	0	2	2	0	3	3
Total	34	34	34	41	41	41

SE group: *N* = 34, NSE group: *N* =41, The others: Non-pretense, Refusal and the other responses. Residual analysis:

† *p* < 0.1,

* *p* < 0.05,

** *p* < 0.01.

The authors apologize for these errors and state that they do not change the scientific conclusions of the article in any way. The original article has been updated.

## Publisher's note

All claims expressed in this article are solely those of the authors and do not necessarily represent those of their affiliated organizations, or those of the publisher, the editors and the reviewers. Any product that may be evaluated in this article, or claim that may be made by its manufacturer, is not guaranteed or endorsed by the publisher.
